# Patient perceptions of foot disability in Juvenile Idiopathic Arthritis: a comparison of the juvenile arthritis foot disability index and the Oxford ankle foot questionnaire for children

**DOI:** 10.1186/s13047-015-0106-5

**Published:** 2015-09-10

**Authors:** Jill Ferrari

**Affiliations:** Department of Health, Sport & Bioscience, University of East London, Stratford, London, E15 4LZ UK; Department of Paediatric Rheumatology, Hospital for Sick Children, Great Ormond Street, London, WC1 UK

**Keywords:** Juvenile idiopathic arthritis, Patient related outcome measures, Foot disability

## Abstract

**Background:**

The Juvenile Arthritis Foot Disability Index (JAFI) and the Oxford Ankle Foot Questionnaire for Children (OxAFQ-C) are two region-specific paediatric outcome tools that measure the impact on well-being in children with foot pathology. The aim of this study was to establish the level of agreement between the JAFI and the OxAFQ-C in a group of children diagnosed with Juvenile Idiopathic Arthritis (JIA).

**Methods:**

Children with JIA accessed the questionnaire via a website. The OxAFQ-C questionnaire and the JAFI questionnaire were combined into one document consisting of 42 statements with Likert-scale responses. A further question regarding duration of disease was added. On completion, the web-linked questionnaire was returned by e-mail.

**Results:**

Thirty five participants were included. Individual domain and composite score analysis was undertaken. The JAFI participation domain was compared to the OxAFQ-C school domain and showed no significant difference between the median scores of each participant (z = -1.33, *p* = 0.181). The JAFI activity and the OxAFQ-C physical domains were compared and showed that a significant difference between the median scores existed (z = -4.29, *p* < 0.001). Agreement between the two PROMs was tested using Bland Altman Levels of Agreement based upon the percentage summed composite scores. Levels of agreement between the scores were considered to be poor based on the Bland Altman plot, despite a low mean difference in scores (mean difference = -3.88, SD of difference = 9.93, *p* = 0.027). Pearson correlation was undertaken to measure the relationship between the summed composite score and disease duration. No relationship was found (JAFI: r = -0.08, *p* = 0.672; OxAFQ-C: r = 0.037, *p* = 0.871).

**Conclusions:**

This study has shown that despite some agreement between the individual domains, overall there is poor agreement between the OxAFQ-C and the JAFI percentage summed composite scores. The study is not able to determine if one score is superior to the other but both scores could be of value when used in this population.

## Background

The podiatrist is likely to encounter arthritic conditions that impact on the feet. Those practitioners treating paediatric foot problems on a regular basis will be familiar with Juvenile Idiopathic Arthritis (JIA) which has a prevalence of approximately 1 in 1000 children in the UK [1]. JIA is known to affect foot joints and other synovial structures causing pain, changes in joint position and functional limitations [2, 3]. As with any arthropathy which affects the foot, it is helpful to determine the impact of the foot problems on the individual. Often, this is determined by simply asking the patient about the foot issues that are of importance to them and returning to these difficulties on follow up appointments, but being a subjective process and influenced by many factors, this method is not ideal. With the development of outcome measures that have been shown to be valid, reliable and sensitive to change, an objective measurement of the impact of the disease on the patient’s well-being is now possible. Validated patient reported outcome measures (PROMs) provide quantifiable data as well as the potential to identify a meaningful change in the condition of the patient. Such PROMs determine the patient’s perception of their foot problems across various aspects of their life. Understanding the patient’s perception allows the treatment to be patient-centred ensuring that the patient drives a goal-orientated approach to the management strategy. Utilising such validated PROMs also allows for ethical and rigorous clinical governance or research. Within podiatry, regional PROMs such as the Foot Function Index (FFI), Foot Health Status Questionnaire (FHSQ) and the Manchester Oxford Foot Questionnaire (MOXFQ) are well known, but these are tools designed to be used with adult patients; children will need outcomes that relate specifically to their daily activities, such as attending school and being able to play. Only two paediatric outcome tools are available that are region-specific and thus measure the impact on well-being of foot pathology in children. One tool is specific to JIA - the Juvenile Arthritis Foot Disability Index (JAFI); the other is a generic tool - the Oxford Ankle Foot Questionnaire for Children (OxAFQ-C). The JAFI was developed in Sweden and the item content was chosen by two physical therapists working in the field, and based on the Foot Function Index and the Sundbom Arthritis Foot Evaluation Index [4]. A sample of seven children and 7 adolescents and their parents reviewed the content of the questionnaire and adjustments were made. Following this, 15 children and adolescents were used in the testing of construct validity, sensitivity and specificity, ceiling and floor effects and reliability. The small sample size involved in the development of the questionnaire limits the robustness of the JAFI. Construct validity was tested by comparing each domain with a different questionnaire. For example, the well-recognised and validated Child Health Assessment Questionnaire (CHAQ) was used to test construct validity of the activity domain. However, the other domains were tested against scales that were made up by the researchers and which thus were not previously validated, undermining the construct validity of the JAFI. Reliability was tested by the small JIA sample being tested a week apart. Reliability of each domain was good, with kappa scores > 0.88, however reliability of individual statements was moderate to poor with the authors considering that the individual scores were impacted by emotional changes of the individual. No ceiling or floor effects were noted. The final questionnaire included 27 statements separated into three domains representing “Impairment”, “Activity Limitation” and “Participant Restriction”. Each of the included statements are answered using a Likert scale graded from Never, Occasionally, Sometimes, Frequently, Always and scored from 0 to 4 respectively so that a high score represents marked impact. The median value for each domain is found and used to identify the impact for each separate area; a composite total score is not suggested. The authors note that the translation of the questionnaire from the original Swedish language has not been validated and sensitivity to change has not been tested.

Being disease-specific, the JAFI does not allow comparisons to be made with other patient groups and if such comparisons are needed then the Oxford Ankle Foot Questionnaire for Children (OxAFQ-C) may be more useful. The OxAFQ-C has been extensively validated for use in a variety of conditions and has been translated and re-validated in several languages, but it has not been used in children with JIA, in published studies. The OxAFQ-C is a robust tool designed for use in children aged 5-16 years to identify the impact on well-being in children with foot problems regardless of the disease or condition involved [5]. The OxAFQ-C was developed initially from focus groups involving parents and then children of differing age groups, to identify consistent themes regarding their foot problems that could be developed into a questionnaire. The JAFI was used in the developmental stages of the questionnaire as a comparison and thus there is some similarities between the two scoring systems. The questionnaire was test on 158 children and factor analysis identified domains that the responses could be grouped into. Face, content and construct validity were tested as well as responsiveness over time and longitudinal validity [5]. Construct validity was tested against other well-recognised validated scales. Also using a 5-point Likert scale, the OxAFQ-C uses the terms Never, Rarely, Sometimes, Very Often and Always. It is graded in the opposite direction from the JAFI, from never (4) to always (0), so that a high score represents minimal impact. The OxAFQ-C consists of 15 statements separated into “School and Play”, “Physical” and “Emotional” domains. A median score is also determined for each domain but the data generated has also been summed for each domain and converted to a percentage score giving mean data for each domain [6]. Again, computing a total composite score is not suggested and as with the JAFI, thresholds for severity have not been published. Minimal detectable change (MDC) has been calculated for the OxAFQ-C and the MDC(90) based upon the 90 % confidence intervals and the standard error of the mean suggested that a value of 6-8 percentage points would indicate change beyond measurement error [6].

Although neither the JAFI nor the OxAFQ-C was developed to be applied as a composite score, a similar adult score - the MOXFQ - has recently researched the use of its domain-based scores as a single composite score [7]. Preliminary research suggests that the single composite score does correlate with other measures of disease impact on well-being and that the benefit of the composite score is that, when used in research studies, it reduces the number of statistical comparisons needed to be performed (unlike testing the individual domains) which in term reduces the role of chance on the hypothesis testing, as well as giving an insight into the overall impact of the condition, with the single figure being easier to interpret than the three individual domains [7].

Although the JAFI should be superior to the OxAFQ-C in children with JIA due to including statements within the questionnaire that focus on the subtleties of the disease, there are in fact only three disease-specific statements: presence of morning stiffness, pain in the morning and presence of joint swelling. Having 27 statements does mean that completion of the score is time-consuming in a clinical setting. Despite the relatively large number of statements, there is some suggestion that the JAFI may have a floor-effect, not being sufficiently sensitive to recognise very mild disease activity [2] although a more recent study did find that the JAFI identified mild-moderate impairment when mild disease activity was present [8]. The OxAFQ-C has evidence to support it as a robust tool to use in paediatric foot problems but whether it can capture the impact of JIA on the feet is not known.

The aim of this study was therefore to establish the level of agreement between the JAFI and the OxAFQ-C in a group of children diagnosed with JIA. This was undertaken using Bland Altman Levels of Agreement [9]. This study also reports the comparisons of medians between the scores for the best matched domains across the questionnaires, although it is recognised that there is no exact match for the domains. Association with disease duration was also considered.

## Methods

The Children’s Chronic Arthritis Association (CCAA) is a UK registered charity providing a national support network for children with JIA and their families. Permission was granted by the CCAA to access children with JIA via their website for this questionnaire-based study. Use of the OxAFQ-C requires permission from ISIS Outcomes at the University of Oxford, and this was granted. Following ethical approval from the University of East London’s School of Health, Sport and Bioscience ethics committee, recruitment of children with JIA was undertaken through the CCAA website. The study was advertised on the CCAA website inviting children and parents to participate. A web-link was provided on the CCAA site which linked to the study website, allowing access to study information and consent forms. Following submission of consent forms and receipt of information to ensure the inclusion criteria (aged 5-16 years old, diagnosis of JIA made by a paediatric rheumatologist and including all subgroups of the disease) were met, the participants were directed to the questionnaire pages on the website, powered by SurveyMonkey Inc.

The OxAFQ-C questionnaire and the JAFI questionnaire were combined into one document consisting of 42 questions. The name of each questionnaire was removed but a break was placed between questionnaires and the participants were invited to take a short rest between the completions of each questionnaire if needed. A further question regarding duration of disease was added to the end of the questionnaire to provide additional information. The questionnaires are appropriate for children to complete alone but parents were asked to supervise the completion and assist as good language skills were required.

To prevent incomplete questionnaire submission, the facility on SurveyMonkey was utilised whereby only full completed questionnaires could be submitted. Completed questionnaires were downloaded and returned via e-mail. All were anonymised prior to data extraction.

### Data analysis

#### Domain analysis

Questionnaire responses were transformed from the 5-point Likert descriptors to their numerical value. The JAFI is graded from never (0) to always (4) thus a higher score indicates greater impact on well-being due to foot disability. However the OxAFQ-C is graded from never (4) to always (0) and thus a lower score indicates greater impact on well-being. To allow direct comparison of the questionnaires, the grading of the OxAFQ-C was reversed (never (0) to always (4)) so that for each questionnaire a higher score represented greater impact. For each domain, the median score was determined. Two of the domains in the JAFI was partnered with the most similar domains in the OxAFQ-C and differences between the domain pairs were considered using Wilcoxon matched-pairs signed-ranks tests.

#### Composite ccore analysis

The data were also considered using the actual summed composite score and the percentage summed composite score. As the summed score for the JAFI is up to a maximum of 108, and the summed score for the OxAFQ-C is up to 60, the data from each score was converted to a percentage score so that each questionnaire had the same unit value. The level of agreement between JAFI and OxAFQ-C scores was assessed using the method described by Bland and Altman considering the mean difference and the 95 % levels of agreement calculated from the standard deviation (1.96*SD of mean difference) [9]. Pearson’s correlation was also used to test for associations between the JAFI and OxAFQ-C percentage composite scores. The questionnaires use a Likert scale to generate the score which is not continuous data as required in Pearson’s correlation, however when each question is totalled to form the actual or percentage summed composite score, the data becomes interval data and thus the assumptions of the Pearson’s correlation are met.

Normality testing determined that the actual summed composite scores and disease duration data were normally distributed thus parametric testing of associations between questionnaires’ actual summed composite score and duration of disease was tested using Pearson’s correlation.

## Results

A total of 35 participants were entered into the study (age range 6 to 16 years). All were diagnosed with JIA, having had the disease for a mean of 4.7 years (range 1 to 11 years). The inclusion criteria was clear on the website and subjects self-assessed that they met these before submitting consent forms. The number of subjects being excluded after consent was not recorded.

### Domain analysis

The median score (and interquartile range) is presented for each of the domains for each questionnaire (see Table 1). Although the JAFI domains and the OxAFQ-C domains were not entirely alike since questions across the domains were not identical, the scores for the JAFI participation domain versus the OxAFQ-C school domain, and the JAFI activity domain versus the OxAFQ-C physical domain were considered to be addressing similar themes and were compared directly using the Wilcoxon matched-pairs signed-ranks test. The impairment and emotional domains were not compared due to marked differences in questions being included in each. The JAFI participation domain and the OxAFQ-C school domain had questions in common that related to school activities and analysis revealed no significant difference between the median scores of each participant (z = -1.33, *p* = 0.181). The JAFI activity and OxAFQ-C physical domains had questions in common that related to difficulty in walking but analysis showed that a significant difference between the median scores existed (z = -4.29, *p* < 0.001).

**Table 1:** Comparison of median scores across the domains between the two questionnaires

JAFI domains median (IQR)		OxAFQ-C domains median (IQR)		Wilcoxon test
Participation	2 (1.5-2.75)	School	2 (1-3)	Z = -1.33, *p* = 0.181
Activity	2 (1-2.5)	Physical	3 (2-3)	Z = -4.29, *p* < 0.001
Impairment	2 (1.5-3)	Emotional	2 (0.25-3)	Not tested

### Composite score analysis

The maximum value for the actual summed composite score for the JAFI (27 statements) is 108; for the OxAFQ-C (15 statements) the maximum value for the actual summed composite score is 60. For the 35 participants, the JAFI mean actual summed composite score was 52.74 (SD = 20.69) and the mean percentage summed composite score was 48.84 (SD = 19.15). For the OxAFQ-C the mean actual summed composite score was 31.63 (SD = 11.39) and the mean percentage summed composite score was 52.71 (SD = 18.99).

**Fig. 1:**
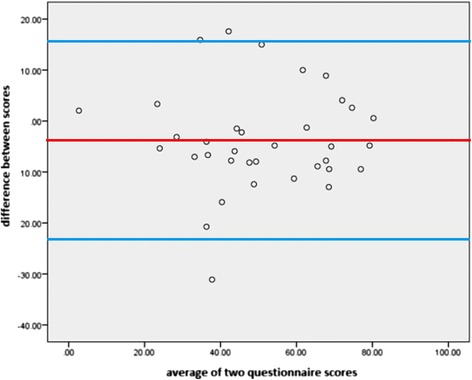
Bland-Altman Plot for Levels of Agreement for the percentage summed composite scores showing mean difference (red line) and 95 % limits of agreement (blue outer lines). Bland-Altman Plot for Levels of Agreement for the percentage summed composite scores showing mean difference (red line) and 95 % limits of agreement (blue outer lines)

Agreement between the two scores was tested using Bland Altman Levels of Agreement based upon the percentage summed composite scores. The Bland-Altman plot (Fig. 1) shows a wide spread of the data with several values close to or just beyond the upper and lower 95 % levels of agreement (ranging from -23.34 to 15.58 percentage points). This identifies that the agreement between the scores was generally poor with agreement between the scores frequently varying despite the fact that the mean difference was small (mean difference = -3.88, SD of difference = 9.93, *p* = 0.027).

A Pearson’s correlation was undertaken between the two percentage summed composite scores which showed a strong association (r = 0.86, *p* < 0.001) but this method of analysis does not show agreement between methods.

### Duration

**Fig. 2:**
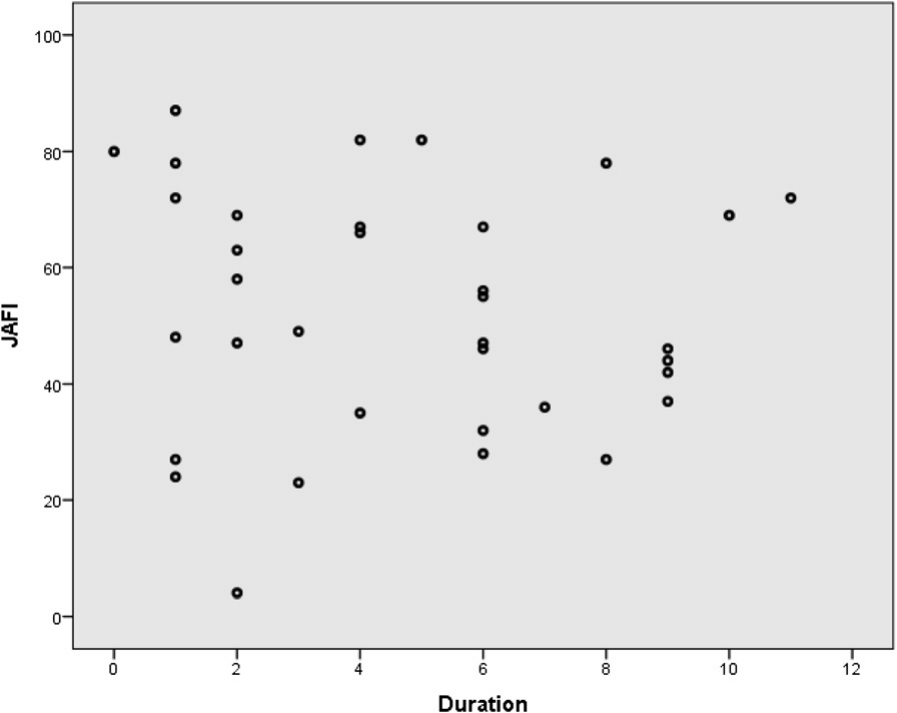
Relationship between JAFI score (actual summed composite) and disease duration (years)

The Kolmogorov-Smirnov Test for normality confirmed the normality of the distribution of the data for the JAFI and OxAFQ-C actual summed composite score and the duration of diagnosis data (KS = 0.104 (*p* = 0.201); KS = 0.11 (*p* = 0.202); KS = 0.15 (*p* = 0.304) respectively). The Pearson correlation was undertaken to measure the association between the summed total score and disease duration. No association was found (JAFI: r = -0.08, *p* = 0.672; OxAFQ-C: r = 0.037, *p* = 0.871). Fig. 2 demonstrates the lack of a relationship between JAFI score and disease duration.

## Discussion

The results have shown that there were varying results when median domain scores were compared between the JAFI questionnaire and the OxAFQ-C questionnaire. When domains were compared that contained questions relating to similar activities, no significant difference in domain scores were seen. This was shown for the JAFI participation domain and OxAFQ-C school domain. The author had expected to find some agreement as the JAFI was used in the development of the OxAFQ-C. However there is limited usefulness in comparing across the domains between the questionnaires as there are clear differences in the themes being probed by the statements. For example, the OxAFQ-C has emotional-themed questions in the emotional domain that considers areas such as being “bothered” by the way the individual walked or how their foot / ankle looked. Such areas are not addressed in the JAFI in a comparable domain as the two emotional-themed statements (being worried or sad about foot problems) are included in the activity limitation domain alongside 11 activity-based questions. Thus the QxAFQ-C emotional domain was not analysed against a JAFI domain in this study. The JAFI activity domain and the OxAFQ-C physical domain were also compared and a significant difference in the median results was found suggesting that the questions asked in each domain were investigating different areas impacting on well-being.

Considering the PROMs from a more general view whereby all the questions are considered without being confined to domains but in a composite score, the study showed that there was a lack of agreement between the questionnaires. Although neither of the questionnaires’ authors suggest the use of a composite score, the OxAFQ-C has been used as a composite score in one previous study [10]. A similar questionnaire, the MOXFQ, has also recently been used as a composite score and this has been recognised as being an acceptable method [7]. The lack of agreement identified between the composite scores of the JAFI and the OxAFQ-C in this study suggests that there are sufficient differences in the two scores such that they are either measuring differing aspects of the perception of well-being through asking different questions or they are identifying different levels of perception. The Bland Altman Levels of Agreement calculation identified a mean difference in summed composite scores of close to four percentage points. The determination of the level of agreement is based primarily on clinical judgement. On initial consideration a mean difference of four percentage between scores would seem to be acceptable clinically. However the Bland Altman plot allows visualisation of how each participant’s scores varied. The plot shows that many participants (14 out of 35) had scores that were more than ten percentage points apart which this author felt would be beyond the clinical acceptability. Although made arbitrarily, this decision was based upon the suggested value of 6-8 percentage points which was the value for meaningful change (MDC) in score for the OxAFQ-C [6]. Several participants had scores with differences close to two standard deviations beyond the mean difference, which is the recommended level used to demonstrate disagreement by Bland and Altman and thus overall, the scores were judged to give differing results. The use of the total composite score has not been investigated in the OxAFQ-C or the JAFI and although a percentage composite score is recommended for each individual domain in the OxAFQ-C, combining all three domains into a single score may mask some important outcomes within the individual domains and “smooth” the overall outcome of the score. Whether this would account for difference between the scores identified in this study is unclear.

The Pearson correlation (r = 0.86) shows that there is a strong association between the scores such that when the JAFI score increases then the OxAFQ-C also increases despite the fact that individual percentage summed composite scores are different. The existence of this association does add strength to the construct validity of each questionnaire which is typically tested by comparing differing questionnaires that test a similar theme.

The data collected in this study is not sufficient to, and did not aim to, identify which questionnaire best reflects the impact on well-being of foot problems in JIA. There have been concerns in past studies that the JAFI was not sufficiently sensitive to identify mild disease having a “floor effect” [2] but since then other studies have shown a strong relationship between disease activity and the JAFI [8, 11]. The JAFI should probably be favoured for use in the JIA population by virtue of the questions that are specific to this condition, such as morning stiffness, morning pain and the presence of joint swelling which should identify when the typical JIA foot pathologies of synovitis, tendinitis and enthesitis are present. But that is not to say that the OxAFQ-C is not useful as it is quicker to complete and has a focus on emotions that may be of value in the teenage population where issues of image can become increasingly important.

When considering the lack of agreement between the scores, the difference in the descriptors may also have played a role in the outcome of this study. Both questionnaires used the descriptors of “never” and “always” to define the end points of the Likert scale, with “sometimes” as the centre value. But the JAFI used descriptors of “occasionally” and “frequently” to complete the range whilst the OxAFQ-C used “rarely” and “very often”. These could have been interpreted differently by the participant and thus lead to the differing scores that have been identified.

A point worth noting regarding the use of the JAFI is that in the activity limitation domain, the meaning of the Likert descriptors is reversed for 11 of the 13 statements. This occurs as the statements change their direction of impact. In the impairment domain the statements are phrased so that the use of the response “always” (score = 4) relates to a poor response so for example “I have morning stiffness in my foot/feet” - a child with a high impact would likely answer “always”. The phrasing changes in the activity impairment domain such that the response of “always” would indicate a good response so for example the statement reads “I can always take part in PE” – if the child answers “always” this would be a good response yet it would still score 4 points. In this study, the scoring was reversed to be consistent with other answers with a high score equating to a greater impact for all statements. Dekker et al [11] also noted this discrepancy and recommended that these questions are adjusted to give consistency across the score.

Despite not suggesting use of the summed composite score for the questionnaires, it would seem to be appropriate to use the composite score when comparing patient groups, whereas the individual domain scores may be more useful for identifying specific areas needing treatment, for example, improving participation in school sports, running ability or addressing footwear concerns. The composite score also allows for a meaningful change in score to be defined. Morris et al [6] suggested that a difference of half the standard deviation of the group would represent a true change beyond the measurement error of the tool. But further work is required in this population in order to determine the minimal clinically important difference (MCID) for each of the PROMs. The MCID is useful to determine the impact of treatment through an improvement in score, but also as a monitoring tool so that any deterioration in outcome beyond the minimally important difference can be recognised and acted on quickly.

Further work on identifying a threshold of impact for the questionnaires would also be useful, perhaps to form a range for “normal” – feet with minimal biomechanical dysfunction and inactive joint disease - through to more severe levels of impact. This would be useful so that the feet considered most at risk from the inflammatory process (with or without biomechanical complications) can be identified and followed closely but also so that any medical professional within the multidisciplinary team can apply the PROM and determine when treatment is indicated and referral to a foot specialist is needed.

Disease duration did not correlate with either questionnaire score. It had been expected that those participants with longer disease duration might show a greater impact of the disease on their feet through their actual summed composite score. However no association was seen and this has also been found in another study [12]. Disease duration is not an indication of disease activity since despite being diagnosed for a long time, the disease may be mild or destructive, well controlled or poorly controlled and this seems to be more dependent on disease subgroup [13] and access to care [14] than duration, thus disease duration was too crude to identify correlations, between severity and the actual summed composite scores, on well-being.

This study was subject to some methodological limitations which might be improved in future studies. As JIA is a condition that can flare and remit and transient pain might occur with increased physical activity, the two questionnaires did need to be complete within a short time of each other. For pragmatic reasons, in this study the questionnaires were completed together but because the process of answering 42 statements may have been arduous, there was the potential that the final statements would not be answered accurately. Using software such as SurveyMonkey does allow observation of the length of time that participants take to complete the questionnaires. For this study, the average time for completion of all statements was 6.7 min (SD = 1.4 mins) which was not as long as anticipated. For future studies it might be useful to format the questionnaires in differing orders for each of the participants to reduce the impact of less care being taken on the final statements. Neither of the questionnaires’ authors reports the need to answer the questions in a set order but it is noted that the emotional statements are at the end of each questionnaire, after the subject has focused on the limitations caused by the condition. This might be important to prepare the subject for the emotional statements and thus rearranging the statements to equalise the potential for rushing the final statements might be inappropriate. The impact of both fatigue in answering questions and the order of the questions in questionnaires is well recognised [15].

Questionnaires such as the JAFI and OxAFQ-C which require the participants to remember events occurring only in the last week, are subject to limitations such as telescoping whereby the participant remembers more dramatic events as being more recent than they actually were. They are also subject to selective recall (confirmation bias) when subjects remember events with confirm the ideas they have already formed. Although these are examples of limitations affecting this type of questionnaire, they are not expected to alter the outcome of the study as they should apply to both questionnaires equally.

There was some small chance that the inclusion criteria for the study were open to abuse. Although only publicised on the CCAA website, this is an open site and it is possible that people other than those with JIA may have chosen to enter the study, or those with JIA, but outside of the inclusion criteria may have entered. Within this study, no exclusion was made for other conditions that might affect the feet such as talipes, tarsal coalition or neurological conditions. Having JIA does not prevent other foot conditions coexisting and therefore, in order to have a sample representative of the normal JIA population, it was decided not to exclude these conditions despite the fact the these conditions are likely to affect the score for each questionnaire.

Both questionnaires are designed for the children from the age of 5 years old to answer on their own. This study did allow the parents to assist the child and therefore the parents may have influenced the outcome of the questionnaires, however it has been recognised that parents are able to rate the consequences of the disease on their child [16]. The decision to allow parents to help was taken as it was felt that the questionnaires were quite complex for the younger children and it also reflected the situation in clinic where even older children would ask parents to help determine the most appropriate answer. The parental influence on both questionnaires would be expected to be equal and therefore not to impact on the results of this study.

## Conclusion

This study has shown that there is a poor level of agreement between the OxAFQ-C and the JAFI when the percentage summed composite score was used, but both scores are likely to be useful in studies on a JIA population or in the clinical setting. Only one of the comparisons between domain scores showed good agreement due to the individual statements within those domains addressing similar themes. It would be useful to establish normal population distributions for JIA for both questionnaires, and future research is needed to identify minimal clinically important difference and thresholds to identify severity.
